# The sucked surgical sponge: Rare case of Gossypiboma after vaginal hysterectomy

**DOI:** 10.1002/ccr3.2074

**Published:** 2019-03-06

**Authors:** Florian Oehme, Annika Rühle, Michael Stickel, Jürg Metzger, Jörn‐Markus Gass

**Affiliations:** ^1^ Departement of Visceral‐, Thoracic‐ and Vascular Surgery University Hospital Dresden Dresden Germany; ^2^ Departement of General Surgery Cantonal Hospital Lucerne Luzern Switzerland

**Keywords:** Gossypiboma, remained surgical material, small bowel obstruction, vaginal hysterectomy

## Abstract

Retained surgical material needs to be a possible differential diagnosis for patients presenting with unspecific abdominal pain after especially cavitary emergency surgery. Even though international standard checklists concerning sponge handling and counting exist, RSM could never be ruled out completely.

## 1 INTRODUCTION

Introducing medical checklists in the very early of this century seemed to be a promising strategy improving patient safety. Due to enhanced communication systems, based on less hierarchical structures, potential complications and structural shortcomings should be detected more frequently.

Especially surgical procedures are known to be on higher risk for structural mistakes as wrong patient or site of surgery, equipment problems and retained surgical material (RSM) inside patients.^1^ The complexity of the surgical procedure and the ongoing handling of information based on the communication with the anesthetist, scrubbing nurse, or surgical assistant during the operation are only some of these influencing factors.

One key element preventing serious adverse events like RSM, as for example Gossypiboma, is the final counting of sponges during abdominal surgery.^2^, ^3^ Gossypiboma, recently described as a well underestimated and underreported medical incidence,^4^, ^5^ refers to the term gossypium (lat. cotton) and the swahili word boma (concealed mass).^6^ RSM is a serious medical problem, which, despite its rare reporting, must be handled as potential differential diagnoses for patients presenting after abdominal surgery with nonspecific abdominal issues.^1^, ^7^, ^8^, ^9^

Primary aim of this case report is therefore, even though standardized procedures and checklist should minimize the risk for RSM, to remind surgeons of Gossypiboma as potential reason for patients presenting with surgical complications.

To the very best of our knowledge, this is the first case report presenting a Gossypiboma after vaginal hysterectomy resulting in a mechanical ileus due to sponge migration into the small bowel.

## 2 CASE HISTORY/CLINICAL EXAMINATION

This case report was written in accordance with the CARE guidelines.^10^

A 80‐year‐old woman presented with history of recurrent vomiting and fatigue to the emergency department of our hospital. Last episode of bowel movement was reported the same day with substantial decrease in quantity. The patient had an episode of fever with up to 38° celsius two days ago. Furthermore, decreased intake of food for the last seven days was mainly due to persisting nausea and vomiting without specific abdominal pain.

Clinical examination revealed a bulging abdomen with crampy epigastric pain radiating to the left lower abdominal quadrant and signs of local peritonism in the right lower quadrant. Sparse bowel sounds were found in each quadrant on auscultation. Further investigations showed a slight elevated heart rate with 91 beats per minute, inconspicuous blood pressure, and temperature. Blood tests indicated a systemic infection with a substantial leucocytosis of 14.7 × 10⁹/L and an elevated c‐reactive Protein (CRP) of 72 mg/L.

Medical history revealed a left mastectomy due to an invasive ductal carcinoma 33 years ago followed by a systemic chemotherapy. The oncological and radiological follow‐up remained without suspicion for recurrency and was terminated years ago.

Due to a descensus vaginalis grade two and a stress incontinence grade three 22 month ago, a vaginal hysterectomy was carried out at our hospital. During the same procedure, a sacrospinous fixation and a reconstruction of the urogenital diaphragm was performed without any peri‐ or postoperative complications.

## 3 DIFFERENTIAL DIAGNOSIS, INVESTIGATIONS, AND TREATMENT

The further diagnostic examinations included an ultrasound and subsequently a triple contrast‐enhanced computer tomography (CT).

### 3.1 Ultrasound

Abdominal ultrasound showed signs of an ileus without clear evidence for a local infection. Dilated small bowel loops up to 2.5 cm with echogenic material within the lumen were seen.

To detect the origin for the small bowel obstruction, we decided to perform a CT scan.

### 3.2 Computer tomography

**Figure 1:**
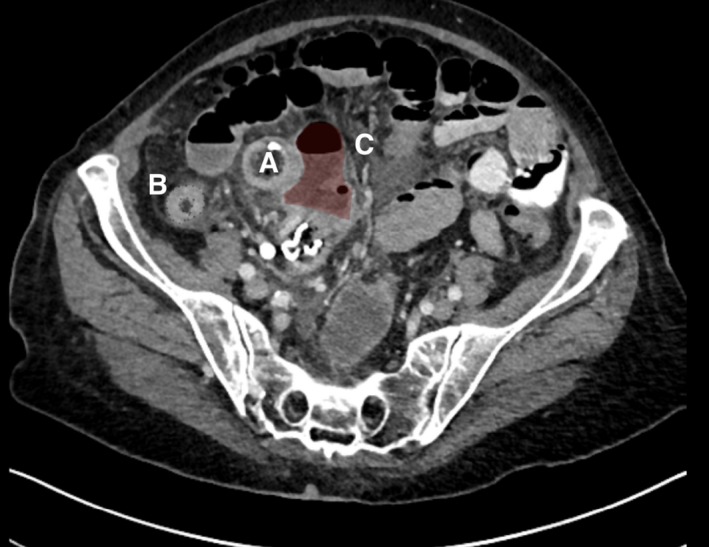
Axial abdominal CT with dilated distal ileum (A), thickened intestinal wall (B), and an interenteric abscess (reddish Zone ‐ C)

**Figure 2:**
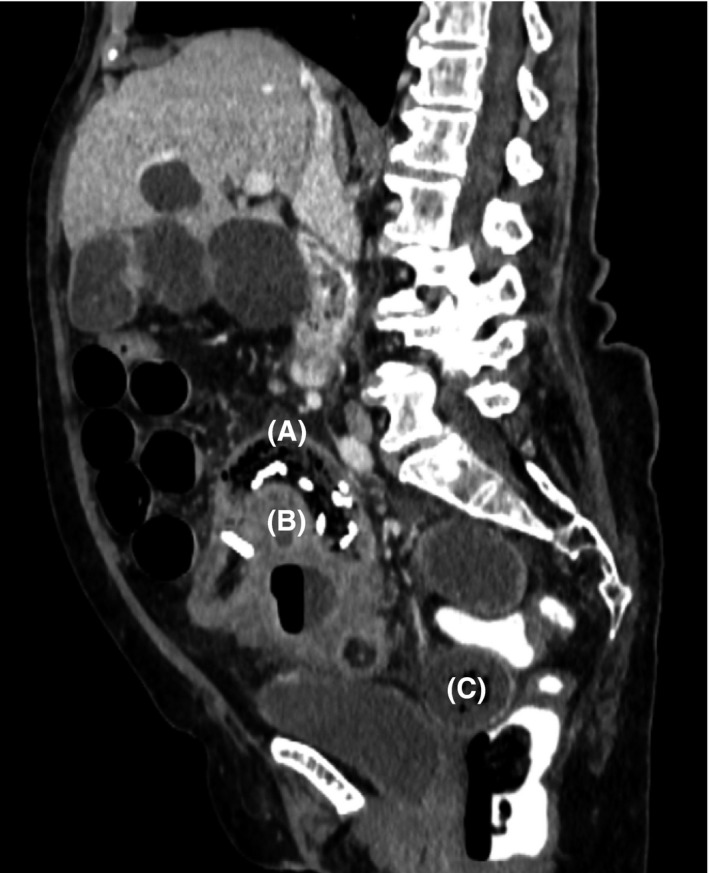
Soft tissue mass with a radio‐opacity line (A) and interenteric abscess (B); small bowel feces sign (C)

A triple contrast‐enhanced CT scan (Figure 1) revealed a 20 cm long segment of dilated distal ileum up to 4 cm with clearly thickened intestinal walls and an interenteric abscess with perforation. Furthermore, signs of a chronically impacted soft tissue mass with a radio‐opacity line were present (Figure 2).

**Figure 3:**
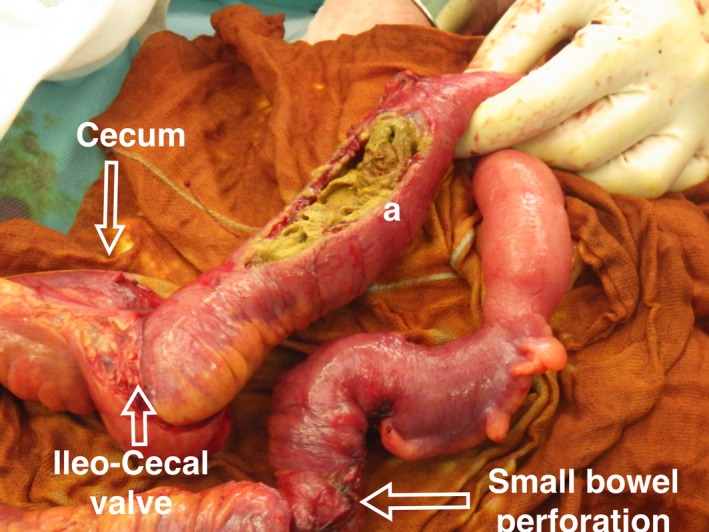
Soft tissue mass (Gossypiboma) after enterotomy

**Figure 4:**
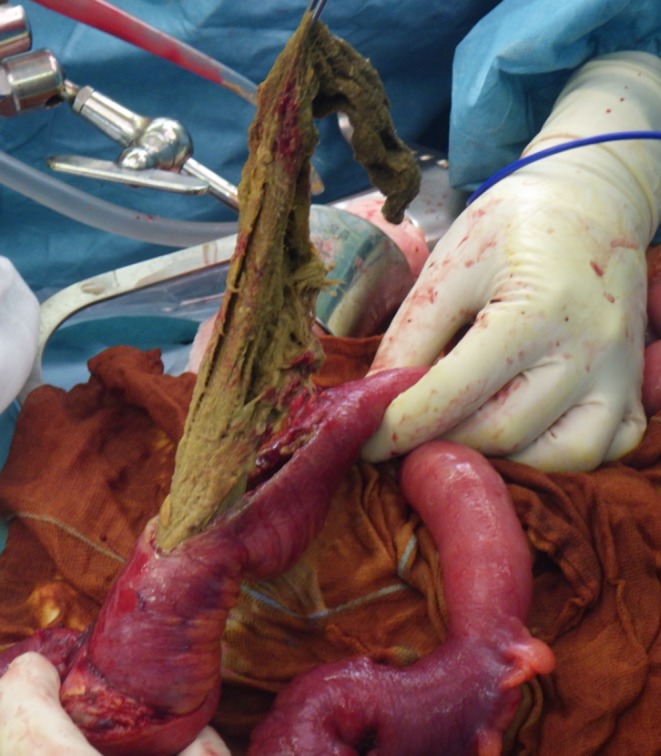
Extraction of the sponge after enterotomy

These findings confirmed a small bowel obstruction due to an intraluminal soft tissue mass accompanied by an interenteric abscess and a small bowel perforation. A RSM was discussed but the intraluminal position of material was misleading. We decided to perform an emergency laparotomy and an enterotomy revealed a surgical sponge that caused the dilation of the small bowel (Figures 3 and 4).

Subsequently, a small bowel resection of 55 cm length was performed followed by saline irrigation of the abdominal cavity. Before ending the operation, we checked for further hollow organ lesions and signs of injuries to the vaginal stump.

We assume that during previous vaginal hysterectomy with sacrospinous fixation and diaphragmatic reconstruction, most probably a surgical sponge was left inadvertently. Due to a chronic inflammatory process, a small bowel perforation around 70 cm prior to the ileocecal valve occured. With the bowel movements, the sponge migrated and caused small bowel obstruction.

## 4 OUTCOME AND FOLLOW‐UP

During the postoperative course persisting high CRP values (90 mg/L) led to another CT scan. A small intra‐abdominal abscess of 4 cm in diameter close to the small bowel was drained and antibiotic therapy administered.

A small wound dehiscence was treated with vacuum assisted dressing (VAC‐therapy), and the patient was discharged 18 days after operation.

Six weeks after the operation, the patient fully recovered with normal frequency of bowel movement, closed laparotomy wound and without further abdominal pain.

The final histological examination performed for the resected small bowel showed a chronic inflammatory process with signs of foreign body reaction. No evidence for malignancy or bacterial infection was seen.

## 5 DISCUSSION

Gossypiboma, as well as retained surgical material in general, is clearly a serious adverse event. Untruthfully, this incidence is believed to be a mistake solely due to inattention that could be fully averted using standardized checklists as suggested by the World Health Organisation.^2^ Thus, RSM was defined as so‐called “never event” in 2009 by the national patient safety agency.^11^

Contrary, with only rare evidence due to a mediocre scientific reporting and an infrequent incidence, the cause for RSM remains most likely multifactorial. The increasing proportion of morbid obese patients, more invasive and complex operating procedures due to scientific advance and multidisciplinary operative approaches are only some that may contribute to an increase for the probability of RSM.^12^

Essential element in preventing RSM, after for example complex emergency operations, is the introduction of checklists with special sections regarding the count of surgical sponges and material. As introduced by the WHO and other scientists, these checklists proved to improve failure frequency in terms of so‐called never events (eg, RSM, wrong site of surgery).^13^, ^14^

Nevertheless, in times when the perspective of policymakers and the society itself is shifting to economization and efficiency, time pressure rises not only for the surgeon but also for the complete operation room team (ORT). Thus, multitasking with distraction of each ORT member due to ongoing communication about further planning or switching between operation rooms (OR) which leads to distraction and the inability to follow the operation in an observant way is resulting. As we know from scientific reports,^12^ exactly these factors are mainly contributing to a flawed counting process. Therefore, we need to acknowledge that surgical counting is a complex process that should be respected as part of the surgical procedure rather than undertaking the effort to educate ORT in terms of counting surgical material on a redundant base. Additionally, ensuring flawless counting with no interruption during the process itself might save a significant amount of hidden costs which could be an even bigger argument for cost efficiency centered perspectives.^15^, ^16^

That RSM is impossible to be ruled out completely was shown by Inaba et al.^1^ They presented during a convincing trial that during 2051 emergency operations involving emergency cavitary surgery, a total of 11 left surgical sponges were detected using a radio frequency detector even though all checklists were fulfilled correctly.

Thus, surgeons need to be aware of the possibility of Gossypiboma, especially during postoperative course after emergency abdominal surgery.

Clinical presentation might range from asymptomatic bulging to septic condition with hollow organ lesion due to persisting low grade infection. In case RSM remains undetected during the early postoperative course, Gossypiboma with fibrinous encapsulation is resulting,^7^ which could possibly be asymptomatic for years. Once the RSM migrate free in the abdominal cavity, a hollow organ perforation could result.

Standard diagnostic procedure should include imaging, as symptoms are not likely to directly link to the diagnosis Gossypiboma. Regularly, ultrasound is the first investigation with only limited value. Subsequently, a triple contrast‐enhanced CT is the diagnostic tool of choice for definitive diagnosis. The CT could display the location of the Gossypiboma using the topogram if a radio‐opaque marker is present. On reconstruction, the CT sequences could possibly display perforations of hollow organ lesion.^7^

In most cases, as the one presented, further investigations could be omitted. Once the diagnosis of Gossypiboma due to RSM is done, definitive treatment includes surgical intervention. In the present case, we decided primarily for a laparotomy. Nevertheless, successful reports of laparoscopic retrieval of RSM can be found in the literature.^17^

## 6 CONCLUSION

Gossypiboma is a serious adverse event that should be omitted. Nevertheless, with increasing complexity during especially abdominal surgery and ongoing time pressure for ORT's as well as increasing BMI in patients, RSM could never be ruled out completely. Indoubtly, minimizing the frequency of RSM should be intended. The counting of surgical material needs to be performed from at least one surgeon and another team member separately. The counting itself has to be a separate stage of the operation that should be accomplished before finishing the operation. Nevertheless, RSM must be taken into account, even years after previous surgery and nonspecific symptoms.

## CONFLICT OF INTEREST

None of the authors or any member of their immediate family has funding or commercial associations (eg, consultancies, stock ownership, equity interest, patent/licensing arrangements, etc) that might pose a conflict of interest in connection with the submitted article. I agree and confirm this statement as true.

## AUTHOR CONTRIBUTION

FO: Contributed to the conception and design of the work, drafting the work, and made the final approval as well as he agreed to be accountable for all aspects of the work; AR: Contributed to the conception and design of the work, drafting the work, revising the manuscript, and made the final approval as well as she agreed to be accountable for all aspects of the work; MS: Contributed to the analysis of the work, drafting the work, and made the final approval as well as he agreed to be accountable for all aspects of the work; JM: Contributed image material, approved the initial idea as solid to construct the case report, interims analysis with further processing ideas were contributed, and made the final approval as well as he agreed to be accountable for all aspects of the work; J‐MG: Contributed to the analysis of the work, drafting the work and made the final approval as well as he agreed to be accountable for all aspects of the work.
